# Surgical Outcomes After Single-Level Endoscopic Transforaminal Lumbar Interbody Fusion: A Systematic Review and Meta-Analysis

**DOI:** 10.7759/cureus.11052

**Published:** 2020-10-20

**Authors:** Courtney E Stone, Brandon L Myers, Sunny Gupta, Tyler X Giles, Neal A Patel, Julian L Gendreau, Mickey E Abraham, Antonios Mammis

**Affiliations:** 1 Neurological Surgery, Mercer University School of Medicine, Savannah, USA; 2 Anesthesia, Eisenhower Army Medical Center, Fort Gordon, USA; 3 Public Health, Emory University School of Medicine - Rollins School of Public Health, Atlanta, USA; 4 Neurological Surgery, Mercer University School of Medicine, Macon, USA; 5 Medicine, Eisenhower Army Medical Center, Fort Gordon, USA; 6 Neurological Surgery, University of California, San Diego, USA; 7 Neurological Surgery, Rutgers New Jersey Medical School, Newark, USA

**Keywords:** general anesthesia, endoscopic, tlif, fusion, transforaminal, lumbar, interbody, eras protocols, local anesthesia, minimally-invasive spine

## Abstract

Background and objective

Novel surgical advancements have introduced endoscopic operative techniques for low back surgery, including transforaminal lumbar interbody fusion (TLIF), which theoretically allows for improved decompression with minimal invasiveness. In addition, endoscopically performed TLIF has allowed for the use of local anesthesia as an alternative method to general anesthesia for patients. We aimed to evaluate the clinical outcomes in patients undergoing endoscopic TLIF and also compare the outcomes in patients undergoing general versus local anesthesia.

Methods

The databases of PubMed, Medline, Embase, and the Cochrane Library were queried for all studies involving patients undergoing endoscopic TLIF. After the extraction of the data and assessment of study quality via the Newcastle-Ottawa Scale, statistical analysis was performed with the R software (The R Foundation, Vienna, Austria) metafor package. The random-effects model was used as the data was largely heterogeneous (I^2 ^>50%).

Results

In total, 15 studies involving a total of 441 patients were selected for the final quantitative meta-analysis. The overall mean difference between the postoperative visual analog scale (VAS) leg scores and preoperative VAS scores was 3.45 (95% CI: 4.93-1.97, p: <0.01). Postoperative VAS low back scores revealed a mean difference of 3.36 (95% CI: 5.09-1.63, p: <0.01). The overall mean difference of ODI scores was 4.58 (95% CI: 6.76-2.40, p: <0.01). Mean blood loss was 136.32 mL and the mean operative time was 149.15 minutes. The mean length of stay postoperatively was lower in the local anesthesia group compared to the general anesthesia group (1.40 vs 5.99 days respectively). There were no outcome variables of patients undergoing general anesthesia versus local anesthesia that showed statistically significant differences in this analysis due to the small amount of data published on patients undergoing endoscopic TLIF with local anesthesia. In addition, the failure of studies in reporting standard deviations as data parameters further limited the quantitative analysis.

Conclusion

Endoscopic TLIF appears to be a viable option for patients undergoing lumbar interbody fusion. Initial data reveal that endoscopic TLIF with local anesthesia may offer patients outcomes similar to those in patients undergoing endoscopic TLIF with general anesthesia, with lower operative times and length of stay.

## Introduction

Lumbar interbody fusion has been used for over a century to treat a variety of neurosurgical conditions, including disc herniation, spinal instability, spondylolisthesis, spinal stenosis, deformity, trauma, malignancy, and infection [1]. First described by Albee and Hibbs in 1911, the fusion of the lumbar spine has been improved by advances in fusion instrumentation, innovative biologics, and new bone-grafting capabilities [1,2]. These advances have expanded the clinical utility of this procedure and greatly improved postoperative outcomes.

Among the potential approaches to the lumbar spine, transforaminal lumbar interbody fusion (TLIF) emerged in the early 1980s and has steadily gained popularity [1,3]. This open approach typically requires a large midline incision with muscle retraction and soft tissue dissection to allow for appropriate visualization, decompression, and spinal instrumentation [3]. Although patients are provided both safe and effective treatment through the traditional TLIF procedure, significant anatomic distortion associated with it is thought to cause lengthy recovery times and increase postoperative pain, blood loss, operative time, and overall healthcare costs [3,4]. As a result, minimally invasive techniques were developed in the 2000s to reduce the incidences of muscle retraction and soft tissue injury [1,3-5].

More recently, novel surgical advancements have enabled further development of endoscopic techniques for TLIF, allowing for improved decompression while achieving minimal invasiveness [6]. Recent literature has supported the efficacy of this novel technique, reporting decreased operative time, length of hospital stay, time to ambulation, and postoperative pain when compared to traditional open TLIF [6,7]. Additionally, endoscopically performed TLIF has allowed the use of local anesthesia as an alternative method to general anesthesia in patients undergoing TLIF with a neuroendoscope.

In this study, we aim to evaluate the surgical outcomes in patients undergoing endoscopic TLIF. Additionally, this study offers a comparison of the surgical outcomes of patients undergoing endoscopic TLIF with general anesthesia versus local anesthesia. To achieve these goals, we performed a thorough and systematic search for studies involving patients undergoing single-level endoscopic TLIF, followed by a meta-analysis of this extracted data.

## Materials and methods

Literature search strategy

This systematic review was conducted according to the guidelines set by the Preferred Reporting Items for Systematic Reviews and Meta-Analyses (PRISMA) [8]. The databases of Medline, PubMed, Embase, and the Cochrane Database of Systemic Reviews were queried with the search phrases (("fusion" and "transforaminal") or ("TLIF")), and ("endoscopic" or "endoscopy")) under all fields. The titles and abstracts of the articles from this search were screened according to the inclusion criteria of this review using the Covidence Systematic Review Management Software (Covidence, Melbourne, Australia). Articles that remained after the initial screening underwent a full-text review for inclusion consideration. The articles referenced in the chosen articles were also reviewed for potential inclusion.

Inclusion criteria

Studies meeting the following selection criteria were accepted for evaluation in this meta-analysis: (1) examined patients undergoing single-level endoscopic TLIF with a clearly defined type of anesthesia, (2) provided data on one or more of the following measures to describe patient outcomes: visual analog scale (VAS) score for low back and/or leg, Oswestry Disability Index (ODI) score, operative time, estimated blood loss, and length of hospital stay.

Exclusion criteria

All literature reviews, case reports, abstracts, and editorials were excluded from this systematic review. Only those papers published within the last 15 years were included. Studies that withheld complete data (values of standard deviation) were excluded from this meta-analysis, as there was no statistical method of accurately incorporating these data without standard deviations. Finally, any studies that did not involve completely endoscopic surgical techniques for single-level interbody fusion were excluded.

Data extraction

All data utilized for the present meta-analysis were derived from the originally published data. There was no process utilized to obtain or confirm this data from primary investigators.

Quality assessment

The quality of the studies was assessed via established Newcastle-Ottawa guidelines [9].

Statistical analysis

The data collection was performed using Microsoft Excel version 16.26 (Microsoft Corporation, Redmond, WA). Meta-analysis was performed using the R software (The R Foundation, Vienna, Austria) metafor package. The random-effects model was used as the data was heterogeneous (I^2^ >50%). Mean difference and a 95% confidence interval were provided for all variables. An α level of 0.05 was used to determine statistical significance. Outcomes were reported as mean differences for pre- and postoperative scoring data, and as mean values for all other data.

## Results

Literature search

**Figure 1:**
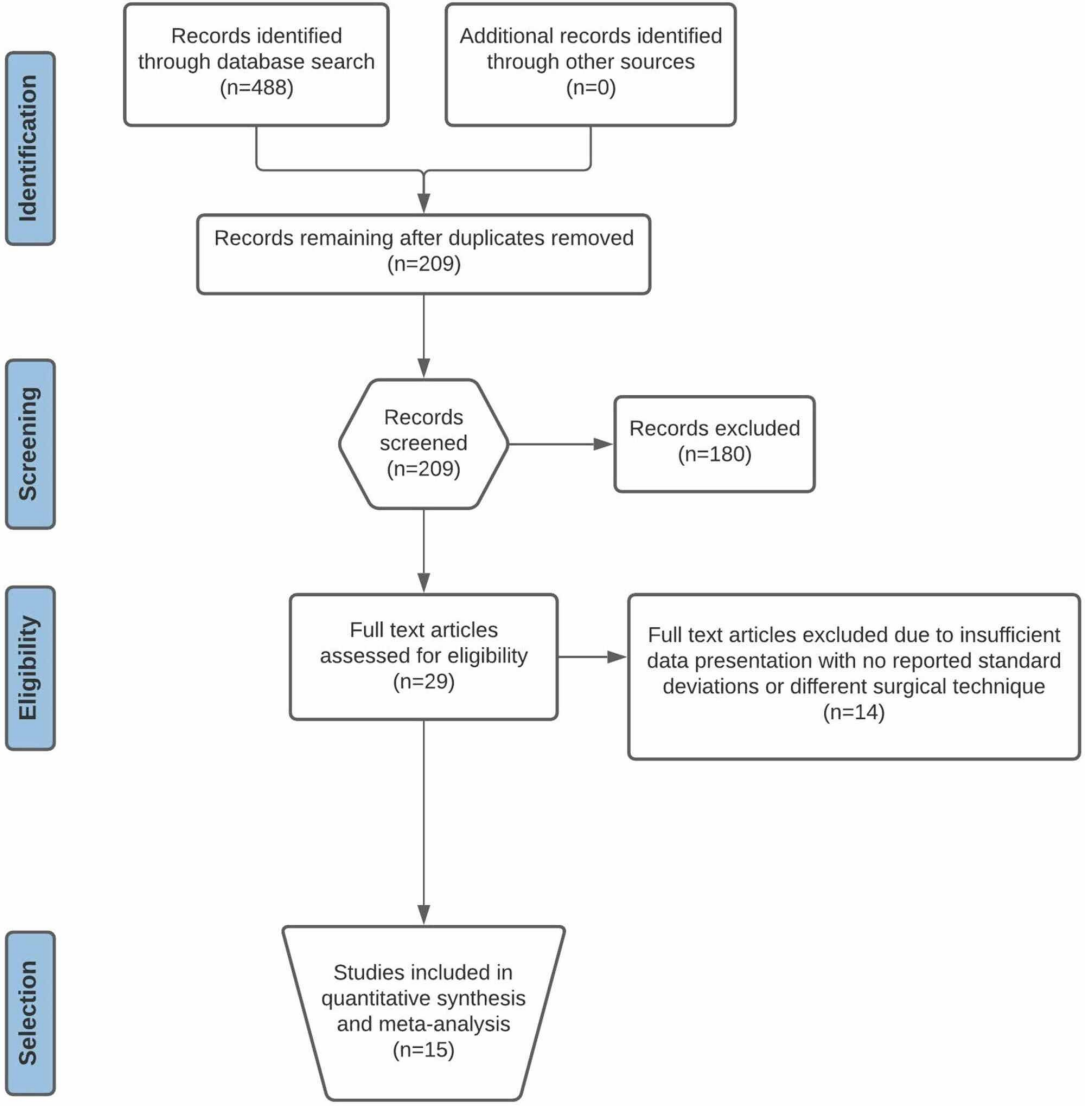
PRISMA flow diagram representing the article selection process for meta-analysis PRISMA: Preferred Reporting Items for Systematic Reviews and Meta-Analyses

The initial database search yielded a total of 155 items from PubMed, 150 from Medline, 179 from Embase, and four from the Cochrane Library of Systematic Reviews. A complete representation of the article selection process is displayed in Figure 1.

In total, 15 studies were selected for the final quantitative meta-analysis in this study [10-24]. A review of the references cited in each of the included studies did not yield any additional studies that met our criteria for inclusion. The characteristics of the chosen studies are summarized in Table 1.

**Table 1:** Characteristics of the selected studies VAS: visual analog scale; ODI: Oswestry Disability Index

Study	Country	Study design	Outcomes measured	Mean follow-up time (months)	Mean operative time (minutes)	Anesthesia type
Ao S et al., 2020 [10]	China	Prospective	VAS leg, VAS low back, ODI, blood loss, length of hospital stay	14	143	General
Kim JE et al., 2018 [11]	Korea	Prospective	VAS leg, VAS low back, blood loss	2	169	General
Lee SH et al., 2017 [12]	Korea	Retrospective	VAS leg, VAS low back, ODI, blood loss, length of hospital stay	46	77	Local
Nagahama K et al., 2019 [13]	Japan	Prospective	Blood loss	22.7	125.4	General
Shen J, 2019 [14]	USA	Retrospective	VAS low back, ODI, blood loss, length of hospital stay	12	168	Local
Wu J et al., 2018 [15]	China	Retrospective	VAS leg, VAS low back, ODI, blood loss	35.1	167.5	General
Yang Y et al., 2015 [16]	China	Prospective	VAS leg, VAS low back, ODI, blood loss	24	178.5	General
Yang J et al., 2019 [17]	China	Prospective	VAS leg, VAS low back, ODI, blood loss, length of hospital stay	15	285.7	Epidural +/- general
Zhang Y et al., 2017 [18]	China	Retrospective	VAS leg, VAS low back, ODI, blood loss	12	174	General
Heo DH et al., 2017 [19]	Korea	Prospective	VAS leg, VAS low back, ODI, blood loss	13.5	165.8	Epidural +/- general
Kolcun JPG et al., 2019 [20]	USA	Retrospective	Blood loss	12	84.5	Local
Heo DH et al., 2019 [21]	USA	Retrospective	VAS leg, ODI, blood loss	12	152.4	Local
Wang MY et al., 2016 [22]	USA	Retrospective	ODI, blood loss, length of hospital stay	12	113.5	Local
Wu J et al., 2019 [23]	China	Prospective	VAS leg, VAS low back, ODI, blood loss, length of hospital stay	12	180.5	General
Wu W et al., 2020 [24]	China	Prospective	VAS leg, VAS low back, ODI, blood loss, length of hospital stay	13.2	184.3	General

Quality assessment

After reviewing the quality of all 15 included articles, 14 articles received a 6 out of a total of 9 possible points and one article received a 5 out of 9 total points. All of the included studies lost one point for failure to select a non-exposed cohort (1 point) and comparability (2 points) since no study described a control group of patients not undergoing fusion. One study lost an additional 1 point for inadequate follow-up [11].

Demographics

The 15 studies evaluated in this meta-analysis comprised a total of 441 patients. The demographic characteristics of the patients are summarized in Table 2.

**Table 2:** Demographics of patients in selected studies

Study	Sample size	Gender (male/female)	Mean age (years)
Ao S et al., 2020 [10]	35	16/19	52.8
Kim JE et al., 2018 [11]	14	6/8	68.7
Lee SH et al., 2017 [12]	18	9/9	44.1
Nagahama K et al., 2019 [13]	25	5/20	68.4
Shen J, 2019 [14]	18	-	66
Wu J et al., 2018 [15]	6	3/3	56
Yang Y et al., 2015 [16]	50	18/32	58
Yang J et al., 2019 [17]	7	1/6	55.3
Zhang Y et al., 2017 [18]	17	6/11	57
Heo DH et al., 2017 [19]	69	24/45	71.2
Kolcun JPG et al., 2019 [20]	84	-	-
Heo DH et al., 2019 [21]	23	7/16	61.4
Wang MY et al., 2016 [22]	10	7/3	62.2
Wu J et al. 2019 [23]	45	13/32	55.98
Wu W et al. 2020 [24]	20	11/9	53.4

Clinical outcome scores

**Figure 2:**
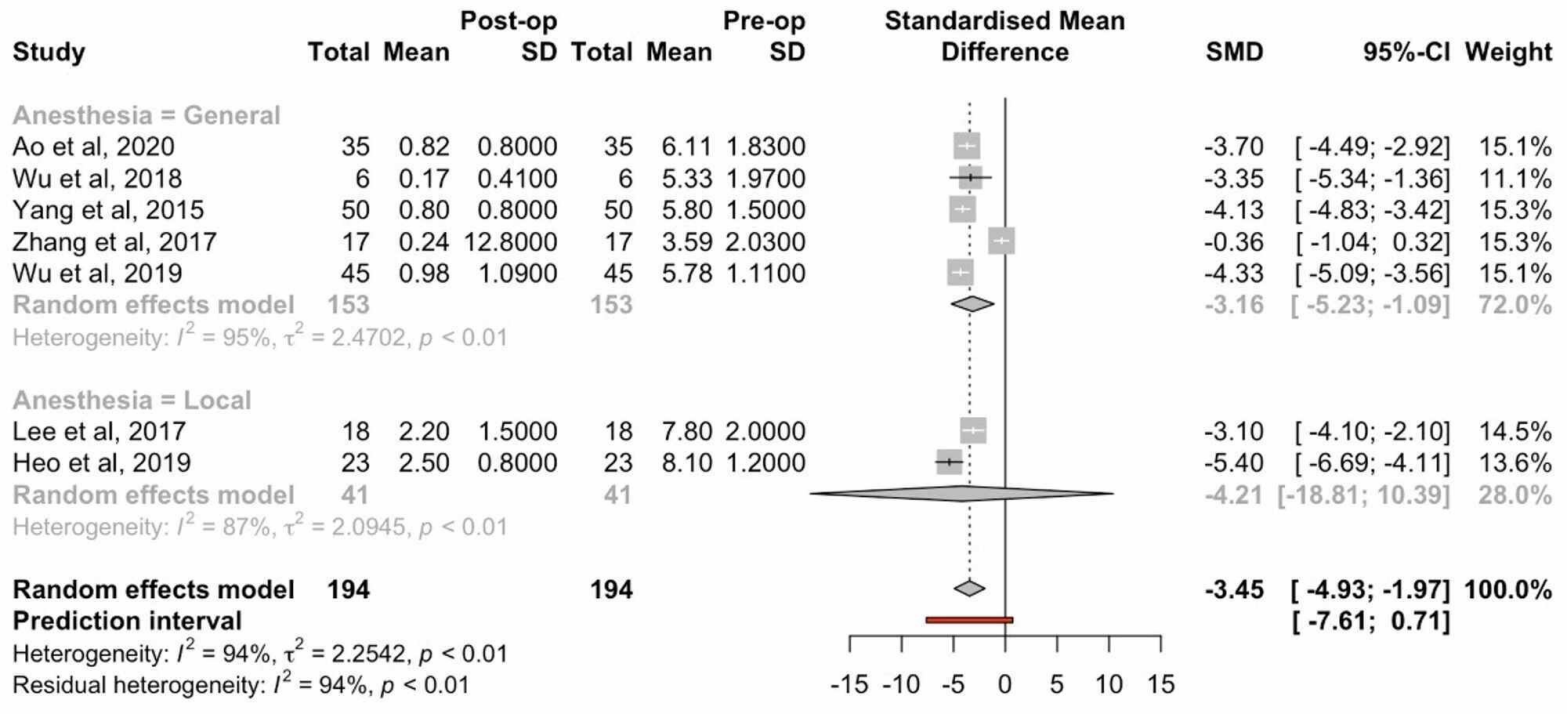
Forest plots comparing preoperative and postoperative VAS leg pain scores VAS: visual analog scale; SD: standard deviation

Several studies provided VAS values for both leg and low back. Three of these studies were excluded based on absent standard deviation values or the failure to distinguish between patients receiving local or general anesthesia. The mean difference between postoperative leg VAS scores and preoperative VAS scores was 3.45 (95% CI: 4.93-1.97, p: <0.01), indicating a statistically significant reduction in VAS leg pain for all patient undergoing endoscopic TLIF. Differences between the cohorts undergoing general anesthesia versus local anesthesia revealed no statistical significance (Figure 2).

**Figure 3:**
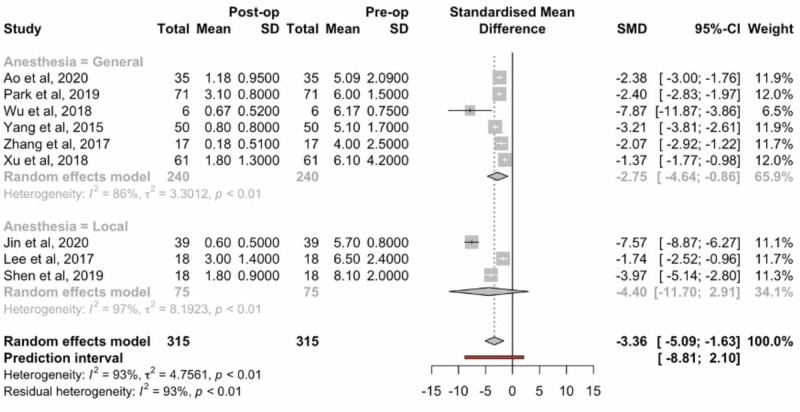
Forest plots comparing preoperative and postoperative VAS low back pain scores VAS: visual analog scale; SD: standard deviation

Analysis of VAS low back scores revealed a mean difference of 3.36 (95% CI: 5.09-1.63, p: <0.01). Statistical analysis revealed no difference between patients undergoing general versus local anesthesia (Figure 3).

**Figure 4:**
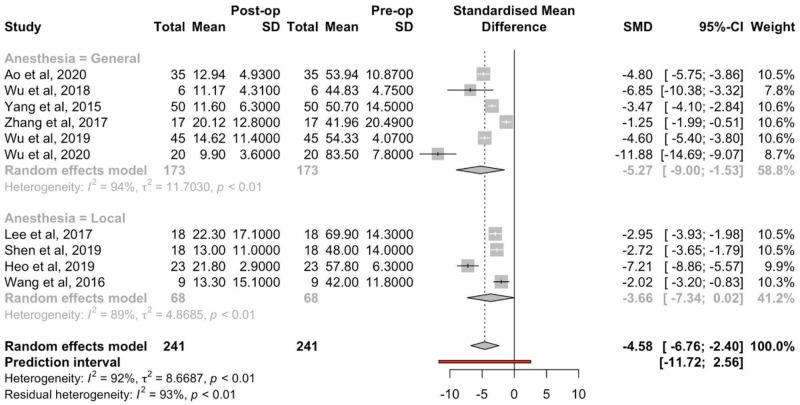
Forest plots comparing preoperative and postoperative ODI scores ODI: Oswestry Disability Index; SD: standard deviation

ODI scores were reported in 12 studies. Two of these 12 studies were excluded from statistical analysis of ODI scores, as patients were not clearly separated by type of anesthesia. The mean difference between postoperative ODI scores and preoperative ODI scores was 4.58 (95% CI: 6.76-2.40, p: <0.01) indicating a statistically significant reduction in ODI score for all patients undergoing endoscopic TLIF (Figure 4). Differences between the cohorts undergoing general versus local anesthesia revealed no statistical significance.

Blood loss

**Figure 5:**
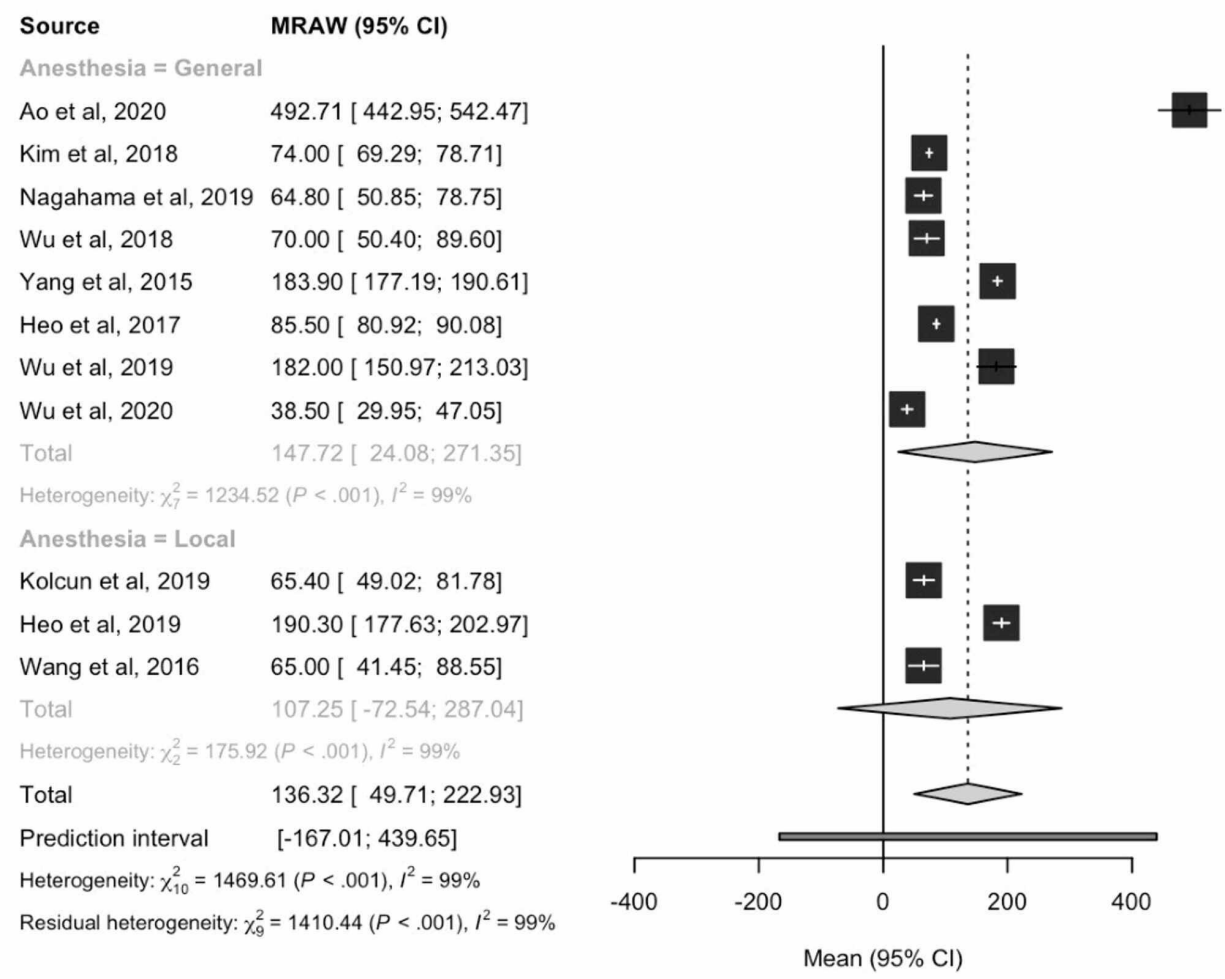
Forest plot comparing mean total blood loss (mL) in patients receiving local or general anesthesia MRAW: the raw data of mean

Overall mean blood loss considering effect sizes was 136.32 mL (95% CI: 49.71-222.93) as determined by 11 studies. The mean blood loss for patients in the general anesthesia group was 147.72 mL, and in patients receiving local anesthesia, it was found to be slightly lower at 107.25 mL (Figure 5). Differences between the cohorts undergoing general versus local anesthesia revealed no statistical significance.

Length of hospital stay

**Figure 6:**
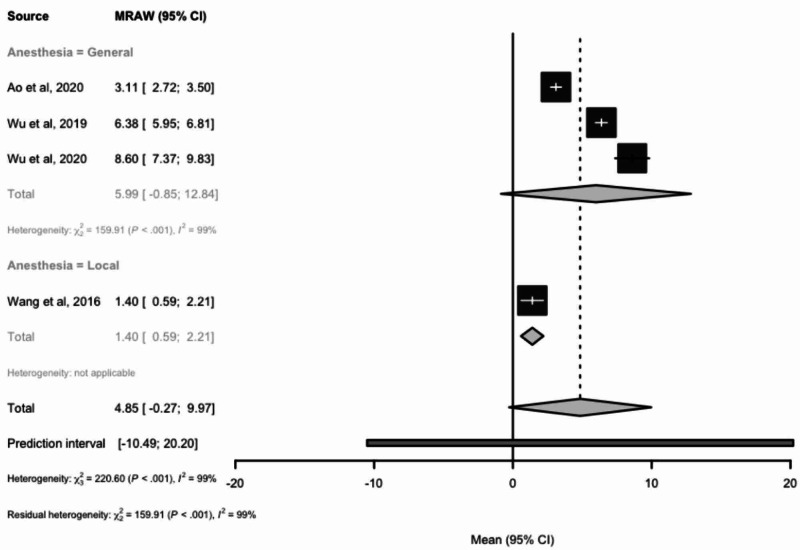
Forest plots comparing the mean length of hospital stay (days) in patients receiving local or general anesthesia MRAW: the raw data of mean

Measurement of mean hospital duration was found to be reported and evaluated in four studies. The mean length of stay considering effect sizes for a total of 110 patients was 4.85 (95% CI: -0.27-9.97) days (Figure 6). The average length of stay was 5.99 days for patients receiving general anesthesia and 1.4 days for patients receiving local anesthesia.

Operative time

**Figure 7:**
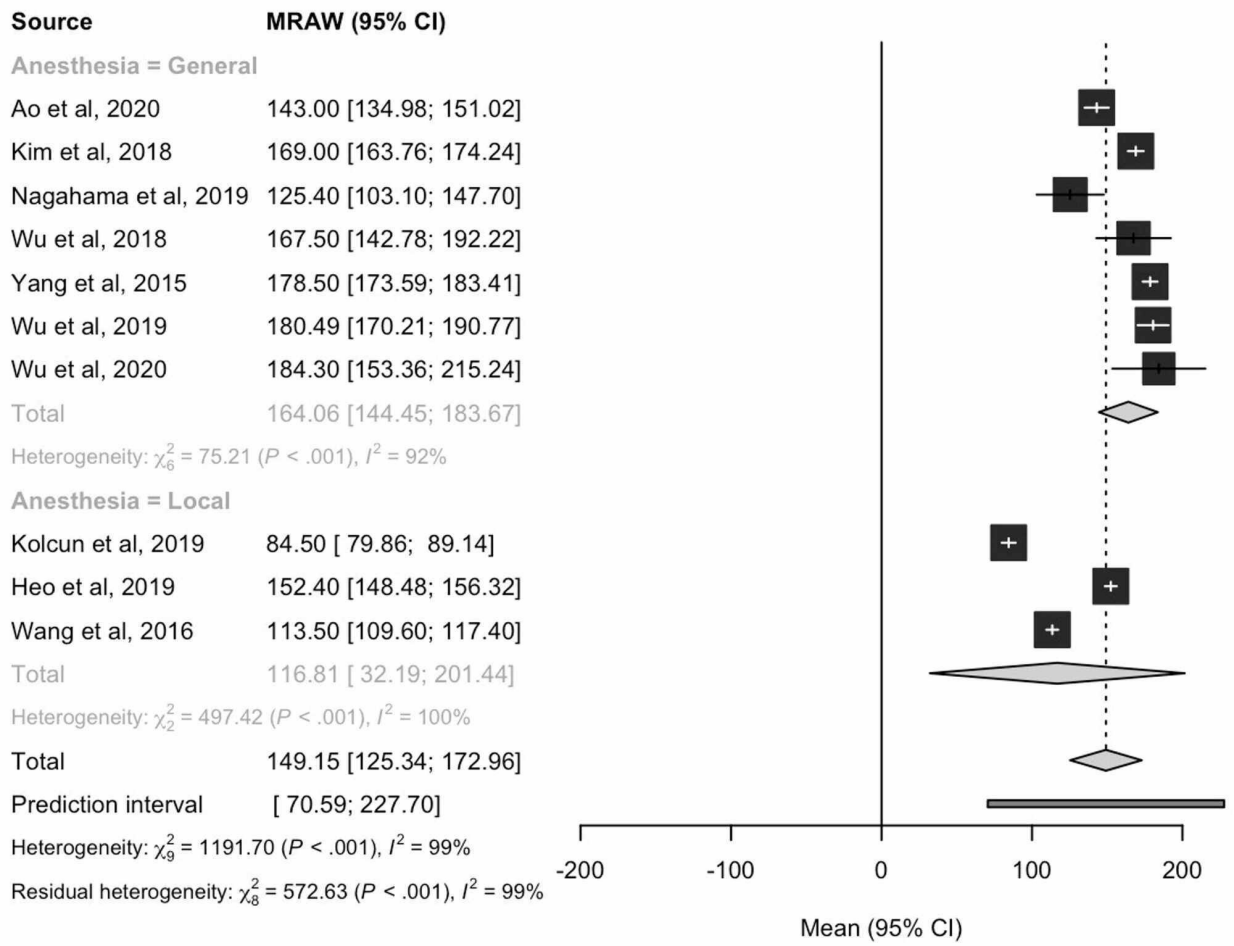
Forest plot comparing mean operative time values (minutes) of patients receiving local or general anesthesia MRAW: the raw data of mean

Mean operative time was analyzed using data from 10 total studies. Mean operative time considering effect sizes was 149.15 (95% CI: 125.34-172.96) minutes. Patients undergoing endoscopic TLIF with local anesthesia were found to have a lower mean operative time of 116.81 minutes than the group treated with general anesthesia with a mean operative time of 164.06 minutes (Figure 7).

## Discussion

The present meta-analysis found a successful reduction in clinical outcome scores postoperatively after patients underwent endoscopic TLIF. It also found that similar surgical outcomes were achieved by either undergoing the procedure with local or general anesthesia. When compared to traditional open TLIF, a meta-analysis by Hammad et al. in 2019 found a mean difference of 4.22 in VAS back pain scores and a mean difference of 4.39 in VAS leg scores postoperatively at similar lengths of follow-up [25]. Our meta-analysis found mean differences of 3.36 and 3.45 for back and leg respectively. In addition, the mean difference was a 22.33 reduction in ODI for open TLIF, while there was a mean difference of only 4.58 in this meta-analysis for endoscopic TLIF. Therefore, there is evidence to suggest that endoscopic fusion may not offer the full utility of benefit when compared to the traditional open surgery at this time.

In the meta-analysis by Hammad et al., the mean blood loss was 568.18 mL in open TLIF. Our study revealed an average blood loss of 136.32 mL in endoscopic TLIF. The length of the hospital stay was 6.92 days for open TLIF, while in this endoscopic study, it was 4.85 days. Additionally, operative time was 198.03 minutes in open TLIF, and it was 149.15 minutes for endoscopic [25]. Therefore, even though it may not achieve similar clinical outcome scores, the endoscopic approach may offer benefits by reducing intraoperative blood loss, patients' hospital stay, and operative time.

When comparing patients treated endoscopically with either local or general anesthesia, this study found that patients undergoing endoscopic TLIF with local anesthesia experienced shorter lengths of hospital stay than those in the general anesthesia group. Unfortunately, this variable did not show statistical significance in this study, likely due to small sample sizes, as endoscopic TLIF is still a relatively novel technique. In addition, many studies retrieved during the systematic search failed to report standard deviations, which further limited the present quantitative analysis.

Operative time was also lower in the local anesthesia group (116.81 compared to 164.06 minutes for the general anesthesia group), but without achieving a statistically significant value. This finding may also be attributable to the small sample size (n=388). It can be inferred that this analysis could have potentially reached a statistically significant level if it had more statistical power. Further, the employment of local anesthesia may lead to improved patient recovery following surgery due to the absence of systemic effects associated with general anesthesia.

Clinical outcome scores for VAS leg pain, VAS low back pain, ODI scores, blood loss, and length of hospital stay were largely comparable between the two anesthesia groups, suggesting that performing this surgery under local anesthesia can result in comparable surgical outcomes. This could prove useful in treating patients with several comorbidities who are unable to tolerate surgery under general anesthesia. It could also be used to treat geriatric patients, as general endotracheal anesthesia has been shown to increase the risk of having longer hospital stays, episodes of readmission postoperatively, acute respiratory failure, and in-hospital mortality in patients aged >60 years [26]. 

In future studies, it is important to elucidate the clinical efficacy of an endoscopic approach to TLIF surgery when compared to open TLIF by carrying out direct comparative trials of the two methods. If spine surgeons can discover ways to improve postoperative surgical outcomes to rates that are comparable with open TLIF, the potential advantages of reduced length of hospital stay and reduced mean operative times can be beneficial to this patient population.

Limitations

The total number of patients included in this meta-analysis was a major limitation, and this was caused by the fact that endoscopic TLIF is a relatively novel modality of fusion that has only recently been devised. While 15 studies were selected for inclusion, almost 14 studies retrieved from the database search met the initial inclusion criteria of patients undergoing endoscopic TLIF but were later excluded. One of the most common reasons for exclusion was that studies reported mean values without standard deviations, which made statistical analysis impossible. Many studies were also excluded because of having a non-clear description of the surgical technique. Future studies evaluating endoscopic spine surgery should aim to clearly report all data values with appropriate standard deviations as well as provide clear descriptions of the surgical technique in order for their data to have maximum utility for future scientific efforts such as systematic reviews.

In addition, the average follow-up time in these studies ranged from two months to 46 months. This wide range of follow-up time is especially important to consider as changes in VAS and ODI scores are directly dependent on the length of postoperative follow-up. 

General endotracheal anesthesia is largely standardized across most medical centers. However, the agents that are used to provide local anesthesia prior to surgery in selected studies may have potentially differed between centers, as this anesthesia modality is not typically employed in spinal fusion cases. It is possible that differences in local anesthetic agents used, dosages, and preoperative waiting times produced changes in mean operative times and/or clinical outcomes measured. Hence, future studies should aim to assess and optimize the standardization of these local anesthetic techniques for endoscopic spine surgery patients.

## Conclusions

Based on our findings, endoscopic TLIF may offer slightly lower clinical outcome scores for patients when compared to traditional open TLIF. No significant differences were found when measuring clinical outcomes scores and mean blood loss between the general and local anesthesia groups. The length of hospital stay was decreased by almost five days and operative time was decreased by almost 50 minutes. Further comparative studies should be performed to directly compare traditional open TLIF versus endoscopic TLIF for the purpose of further assessing the efficacy of the endoscopic approach versus the traditional open surgery.
